# Correction: Genomic surveillance uncovers a pandemic clonal lineage of the wheat blast fungus

**DOI:** 10.1371/journal.pbio.3002236

**Published:** 2023-07-19

**Authors:** Sergio M. Latorre, Vincent M. Were, Andrew J. Foster, Thorsten Langner, Angus Malmgren, Adeline Harant, Soichiro Asuke, Sarai Reyes-Avila, Dipali Rani Gupta, Cassandra Jensen, Weibin Ma, Nur Uddin Mahmud, Md. Shabab Mehebub, Rabson M. Mulenga, Abu Naim Md. Muzahid, Sanjoy Kumar Paul, S. M. Fajle Rabby, Abdullah Al Mahbub Rahat, Lauren Ryder, Ram-Krishna Shrestha, Suwilanji Sichilima, Darren M. Soanes, Pawan Kumar Singh, Alison R. Bentley, Diane G. O. Saunders, Yukio Tosa, Daniel Croll, Kurt H. Lamour, Tofazzal Islam, Batiseba Tembo, Joe Win, Nicholas J. Talbot, Hernán A. Burbano, Sophien Kamoun

The thirteenth author’s name is misspelled. The correct name is: Md. Shabab Mehebub.
