# Supplementary material for: Genomic surveillance uncovers a pandemic clonal lineage of the wheat blast fungus
Source: PLoS Biol. 2023 Apr 11;21(4):e3002052. doi: 10.1371/journal.pbio.3002052 (PMC10089362; doi:10.1371/journal.pbio.3002052)
Supplement: Attachment — Submitted filename: Response_to_Editor_and_Reviewers.pdf [file pbio.3002052.s021.pdf]

RESPONSE TO EDITOR

Dear Sophien,

Thank you for your patience while your manuscript "A pandemic clonal lineage of the wheat blast fungus" went through peer-review at PLOS Biology. Your manuscript has now been evaluated by the PLOS Biology editors, an Academic Editor with relevant expertise, and by several independent reviewers. I'm handling this paper temporarily while my colleague Dr Paula Jauregui is out of the office.

In light of the reviews, which you will find at the end of this email, we are pleased to offer you the opportunity to address the comments from the reviewers in a revision that we anticipate should not take you very long. We will then assess your revised manuscript and your response to the reviewers' comments with our Academic Editor aiming to avoid further rounds of peer-review, although might need to consult with the reviewers, depending on the nature of the revisions.

IMPORTANT: Please address the following:

a) Please could you change the title to something a bit more declarative? We suggest something like: "Genomic surveillance identifies a pandemic clonal lineage of the wheat blast fungus"

We have changed the title following your suggestion:

“Genomic surveillance uncovers a pandemic clonal lineage of the wheat blast fungus”.

b) Please attend to the requests from the reviewers.

Below, you can find a point-by-point reply to the reviewers.

c) Please ensure that you comply with our Data Policy requests; specifically, we need you to supply the numerical values underlying Figs 1ABC, 2ABC, 3C, 4A, S1, S2, S3, S4, S5, S6, S7AB, S8AB, S9, S10, S11, S12CD, S14 (some of

these will be treefiles, I guess, rather than numbers), either as a supplementary data file or as a permanent DOI'd deposition. I note that some of these data may be in your GitHub deposition (https://github.com/Burbano-Lab/wheat-clonal-linage); if so, please clarify, and supply a DOI'd version (e.g. in Zenodo, Figshare, etc.)

We provide now numerical values for each main and supplementary figure through a Github deposition (https://doi.org/10.5281/zenodo.7590238).

d) Please cite the location of the data clearly in all relevant main and supplementary Figure legends, e.g. "The data underlying this Figure can be found in S1 Data" or "The data underlying this Figure can be found in https://doi.org/XXXX"

We added the sentence "The data underlying this Figure can be found in https://doi.org/10.5281/zenodo.7590238" to all figure legends.

We expect to receive your revised manuscript within 6 weeks. Please email us (plosbiology@plos.org) if you have any questions or concerns, or would like to request an extension.

At this stage, your manuscript remains formally under active consideration at our journal; please notify us by email if you do not intend to submit a revision so that we withdraw the manuscript.

IMPORTANT - SUBMITTING YOUR REVISION

Your revisions should address the specific points made by each reviewer. Please submit the following files along with your revised manuscript:

1. A 'Response to Reviewers' file - this should detail your responses to the editorial requests, present a point-by-point response to all of the reviewers' comments, and indicate the changes made to the manuscript.

*NOTE: In your point-by-point response to the reviewers, please provide the full context of each review. Do not selectively quote paragraphs or sentences to reply to. The entire set of reviewer comments should be present in full and each specific point should be responded to individually.

You should also cite any additional relevant literature that has been published since the original submission and mention any additional citations in your response.

2. In addition to a clean copy of the manuscript, please also upload a 'track-changes' version of your manuscript that specifies the edits made. This should be uploaded as a "Revised Article with Changes Highlighted " file type.

Resubmission Checklist
When you are ready to resubmit your revised manuscript, please refer to this resubmission checklist: https://plos.io/Biology_Checklist

To submit a revised version of your manuscript, please go to https://www.editorialmanager.com/pbiology/ and log in as an Author. Click the link labelled 'Submissions Needing Revision' where you will find your submission record.

Please make sure to read the following important policies and guidelines while preparing your revision:

Published Peer Review
Please note while forming your response, if your article is accepted, you may have the opportunity to make the peer review history publicly available. The record will include editor decision letters (with reviews) and your responses to reviewer comments. If eligible, we will contact you to opt in or out. Please see here for more details:
https://blogs.plos.org/plos/2019/05/plos-journals-now-open-for-published-peer-review/

PLOS Data Policy
Please note that as a condition of publication PLOS' data policy (http://journals.plos.org/plosbiology/s/data-availability) requires that you make available all data used to draw the conclusions arrived at in your manuscript. If you have not already done so, you must include any data used in your manuscript either in appropriate repositories, within the body of the manuscript, or as supporting information (N.B. this includes any numerical values that were used to generate graphs, histograms etc.). For an example see here:

http://www.plosbiology.org/article/info%3Adoi%2F10.1371%2Fjournal.pbio.1001908#s5

Blot and Gel Data Policy
We require the original, uncropped and minimally adjusted images supporting all blot and gel results reported in an article's figures or Supporting Information files. We will require these files before a manuscript can be accepted so please prepare them now, if you have not already uploaded them. Please carefully read our guidelines for how to prepare and upload this data: https://journals.plos.org/plosbiology/s/figures#loc-blot-and-gel-reporting-requirements

Protocols deposition
To enhance the reproducibility of your results, we recommend that if applicable you deposit your laboratory protocols in protocols.io, where a protocol can be assigned its own identifier (DOI) such that it can be cited independently in the future. Additionally, PLOS ONE offers an option for publishing peer-reviewed Lab Protocol articles, which describe protocols hosted on protocols.io. Read more information on sharing protocols at https://plos.org/protocols?utm_medium=editorial-email&utm_source=authorletters&utm_campaign=protocols

Thank you again for your submission to our journal. We hope that our editorial process has been constructive thus far, and we welcome your feedback at any time. Please don't hesitate to contact us if you have any questions or comments.

Sincerely,

Roli

Roland Roberts PhD
Senior Editor
PLOS Biology
rroberts@plos.org

on behalf of

Paula Jauregui, PhD,
Editor
PLOS Biology
pjaureguionieva@plos.org

RESPONSE TO REVIEWERS' COMMENTS:

Reviewer #1:
[identifies themself as Johanna Rhodes]

This paper addresses the threat of wheat blast disease, which threatens global food security, and includes work that I believe is incredibly novel on at the forefront of this field, by combining genomics and laboratory experiments. I only have a few comments that I believe, if addressed, would make this article even stronger.

We are thankful for Reviewer 1's enthusiasm and very positive assessment of our work. Their thoughtful comments have contributed to improving the quality and readability of our manuscript. We address these comments below and refer to page and line numbers when we modified the text.

For the introduction, could you expand on the sentence explaining the importance of carrying the RWT4 gene, but not RWT3, and how this ties in with the PWT3/4 effectors?

We have modified the sentence and now we explicitly explain the recognition of PWT3 and PWT4 by RWT4 and RWT3, respectively.

"Wheat blast emerged in Brazil in 1985 following the widespread deployment of wheat genotypes carrying the *RWT4* resistance gene but lacking *RWT3* (*RWT4+*/*RWT3-*). These two resistance genes recognize the blast effectors PWT3 and PWT4, respectively. Thus, the deployment of RWT4+/RWT3-

varieties facilitated host jumps of *M. oryzae* isolates carrying PWT3, but not PWT4 effectors from ryegrass (*Lolium* spp.) to wheat, which was followed by loss of function mutations in the PWT3 effector and subsequent spread to common wheat varieties (6)."

Methods:

Some more clarity on the 84 diagnostic SNPs would be helpful; I looked at the referenced paper in the methods (article 7, "Emergence of wheat blast in Bangladesh was caused by a South American lineage of Magnaporthe oryzae"), yet I couldn't see how the 84 SNPs were derived. As such, a brief description describing how the genotypes were extracted, and what these diagnostic 84 SNPs are would be helpful, as at the moment I can't see how they are informative for discriminating between lineages and deriving ancestral origins.

The relevant preprint that describes the multiplex amplicon sequencing dataset isn't the one the reviewer refers to, but rather: Tembo et al. Multiplex amplicon sequencing dataset for genotyping pandemic populations of the wheat blast fungus Zenodo https://doi.org/10.5281/zenodo.4605959

We now clarified in the material and methods how the 84 SNPs were originally ascertained and how the diagnostic SNPs were extracted from available genome data:

"Selection of SNP panel for multiplex amplicon sequencing

To identify the SNPs that could be used for genotyping of M. oryzae from Bangladesh by multiplex amplicon sequencing (Floodlight Genomics, https://floodlightgenomics.com), we filtered 15,871 SNPs identified in Islam et al. (2016) using the following criteria: (i) they had to be polymorphic among wheat blast strains alone to make the markers useful for diagnostics; (ii) the minor allele was >30%; (iii) SNPs were located on long exonic sequences (> 1500 bp without interrupting intron); (iv) long exons to contain only 2-4 SNPs. The last two criteria were to make sure that the assay will focus on SNPs surrounded by well-conserved stretches among wheat blast strains with an aim to reduce

amplification failures due to polymorphism in the primer binding sites. The above criteria reduced the available genomic regions to 102 loci. We designed 102 PCR primer pairs to amplify ~200 bp amplicon for each gene containing 100 bp flanking regions on each side of the SNP locus for multiplex amplicon sequencing. Trial sequencing runs with these primer pairs resulted in 84 primer pairs that consistently produced amplicons that can be sequenced to identify the correct nucleotides in those SNP loci in Bangladeshi wheat blast isolates (Win et al., 2021). We used these as a panel of 84 SNPs to discriminate between the wheat blast clonal lineage of M. oryzae in Bangladesh from other genotypes. Initial analyses and the use of the 84 SNP panel was reported in Win et al. (2021), and the details of 84 SNPs including gene names and primer sequences along with the dataset for benchmarking are available in Tembo et al. (2021). "

It might be that this has been done elsewhere, and hasn't been sufficiently referenced in this paper; but if not, benchmarking how these 84 SNPs are indeed informative in comparison to the whole genome, would aid this part of the paper considerably.

To show that the set of 84 SNPs are informative, we have now included a new analysis, in which we compared the genetic distances between each pair of blast isolates using the set of 84 SNPs, and the genome-wide SNPs. Our analysis revealed a correlation of 0.82 between the two sets of pairwise distances (Figure SXC). To show that this correlation is robust and much higher than expected by chance, we repeated the calculation of pairwise distances for both datasets (84 SNPs and genome-wide SNPs) randomly subsampling a subset of the isolates (10%) with and without isolate names permutation 100 times. This analysis reveal a median correlation coefficient of 0.82. Similarly, we randomly permuted distance values and sample pairs. We recalculated the correlation coefficients and obtained a median value of 0.001 (Fig. SXD).

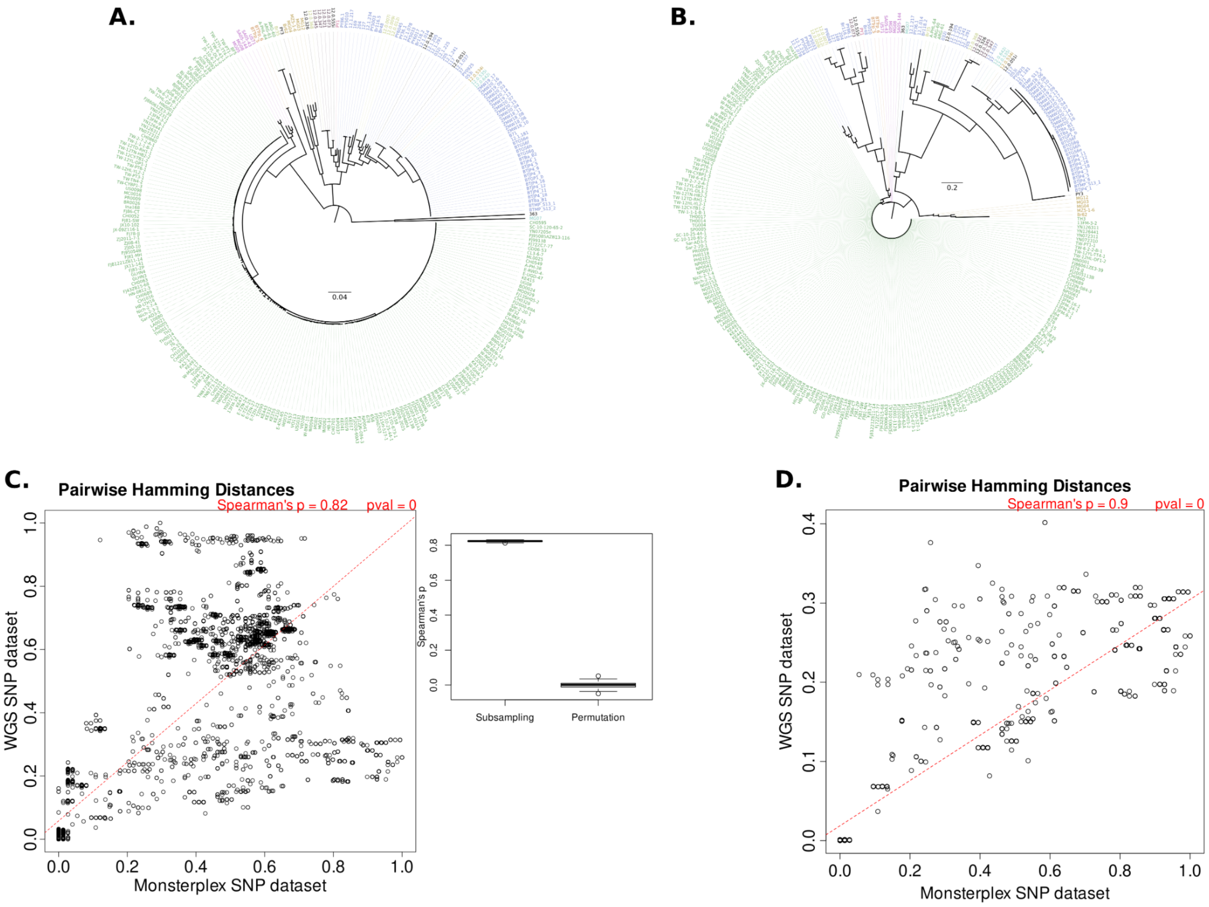

Figure S2. The set of 84 Monsterplex SNPs reflects the patterns of genome-wide diversity of the blast fungus. Neighbor-joining tree of 284 worldwide distributed *M. oryzae* isolates based on 84 concatenated SNPs (**A**) or genome-wide SNPs (**B**). **(C)** The scatter plot shows genetic distances between each pair of blast isolates using the set of 84 SNPs, and the genome-wide SNPs. The boxplots show the correlations of genetic distances between each pair of isolates using using the set of 84 SNPs, and the genome-wide SNPs. The distributions were generated by randomly subsampling a subset of the isolates (10%) with and without isolate names permutation 100 times. **(D)** The scatter plot shows pairwise genetic distances including only the wheat-infecting blast isolates for the set of 84 SNPs, and the genome-wide SNPs.

We included a sentence in the main text highlighting these new results and the corresponding section in the supplementary materials:

“We genotyped 537 *M. oryzae* samples from different geographical regions and hosts based on multiplex amplicon sequencing (MonsterPlex; see material and methods) (N=237) and publicly available genomes (N=351) (Fig. 1, Fig. S1, Table S1). Using the set of isolates from which we genotyped the 84 SNPs and also sequence their whole genomes, we showed that the set of 84 Monsterplex

SNPs accurately reflects the patterns of genome-wide diversity and host specificity of the blast fungus. (Fig. SX)"

"To show that the set of 84 SNPs are informative, we compared the genetic (Hamming) distances between each pair of blast isolates using the set of 84 SNPs, and the genome-wide SNPs. Our analysis revealed a correlation coefficient of 0.82 between the two sets of pairwise distances (Figure S2C). To show that this correlation is robust and much higher than expected by chance, we repeated the calculation of pairwise distances for both datasets (84 SNPs and genome-wide SNPs) randomly subsampling a subset of the isolates (10%) with and without isolate names permutation with 100 repetitions. This analysis revealed a median correlation of pairwise distances of 0.82 and 0.001 for the resampling with permuted distances and sample pairs (Fig. S2C). We repeated the analysis using only pairwise distances among wheat-infecting isolates and obtained a correlation coefficient of 0.9 (Fig. S2D), which shows that the set of 84 SNPs accurately reflect the genetic diversity of the wheat blast fungus"

There is mention of missing sites being removed; including missing positions, but labelling them as such, can be informative. Missing positions could be missing due to sequencing/PCR error or removed doing the bioinformatics analysis; this does not mean the position is not there, just that you are not certain it's there to high confidence. Including missing positions, and setting them to missing would be much closer to the biology.

There are two steps in our analysis, in which we report missing/filtered positions. In both cases, we provided files where the missing/filtered positions can be identified. In the section of "Processing of short reads and variant calling", the missing/filtered positions can be identified in the Variant Call Format (VCF) files, whereas in the section "Phylogenetic analyses, estimation of evolutionary rates and divergence times", they are reported in the XML files that were the input for the BEAST phylogenetic analysis. All the above-mentioned files are available through our Github deposition (https://doi.org/10.5281/zenodo.7590238).

Finally, regarding the temporal analysis using BEAST: whilst HKY is less complex, this might not necessarily be the best fit for your data. A better approach would be to use bModelTest (under different combinations of demographic and clock scenarios) to assess which is the best model to use.

The reviewer suggests a combinatorial approach, in which multiple clocks and demographic scenario combinations are tested under different evolutionary models. This approach will be computationally time-consuming and very likely not necessary. Below we explained point-by-point our approach to handling the substitution model, the demographic scenario, and the molecular clock:

Substitution model: As explained in the text we used HKY, which is a simple substitution model, that only parametrizes exchangeability parameters for transitions and transversion and base frequencies. We followed the suggestion of Nascimento, Do Reis and Yang, Nature Ecology and Evolution - 2017 (ref. 55). They stated that different substitution models tend to give similar sequence distance estimates when sequence divergence is less than 10%, which is our case. Thus, it is better to use a simple model. The same publication suggests that when dealing with shallow divergences, as in our case, there are not enough substitutions to reliably estimate multiple parameters. Complex substitution models are more appropriate for deep evolutionary times.

To illustrate this point we repeated the BEAST analysis using GTR, a more complex substitution model, in which all exchangeability parameters (every single transition and transversion) and base frequency parameters need to be estimated. We retrieve the same tree topology and extremely similar evolutionary rates (HYK 95CI: 6.21e-7 - 7.58e-7; GTR 95CI: 6.23e-7 - 7.54e-7). However, the effective sample sizes (ESS) of the evolutionary rate were lower using the GTR than in our HKY original analysis, 168 and 509, respectively. We have included this new analysis in the material and methods.

Demographic scenarios: as explained in the manuscript, to avoid making and testing multiple demographic history assumptions, we selected an Extended Bayesian Skyline approach (ref. 56). Our approach reduces the demographic assumptions and the computing resources needed for the analysis.

Molecular clock: we now include a new analysis, in which we ran BEAST2 analysis using the HKY substitution model but this time with a random local clock model, which considers whether each branch in the tree needs its own branch rate. We obtained evolutionary rates extremely similar to our original

estimates (Strict clock 95CI: 6.21e-7 - 7.58e-7; Random local clock: 95CI: 6.22e-7 - 7.56e-7). We have included this new analysis in the manuscript.

All in all, our estimated evolutionary rates are robust to the choice of both substitution and clock models and make no assumptions regarding the demographic scenario.

We modified the main text:

“We removed regions that disrupt the clonal pattern of inheritance (Fig. S6-S78) [13] and tested for a correlation between genetic distances and collection years (Fig. S9). We obtained rates ranging from 2.74e-7 to 7.59e-7 substitutions/site/year (Table S3), which were robust to the choice of both substitution and clock models (Table S4).

We modified the material and methods and include two additional supplementary tables:

“To test the robustness of our evolutionary rate estimation to changes in substitution and clock models, we repeated the analysis using GTR in combination with a strict clock model, and HYK in combination with a random local clock model.”

The dates of emergence need confidence intervals (in the Results section), and the rates seem very small - how do these compare to other rates in fungi?

The confidence intervals for the emergence times and evolutionary rates can be found in the results section: “*...we dated the emergence of the Asian and African sub-lineage to similar periods (2009-2012 and 2010-2015, respectively)...*”.

The evolutionary rate that we calculated for the wheat blast fungus is indeed faster than rates previously calculated by us (Latorre et al., 2020, BMC Biology) and others (Gladieux et al., 2018, mBio) for the rice blast fungus. It is important to highlight that the wheat blast fungus evolutionary rate we present in the manuscript was calculated using outbreak data. It has been shown before, theoretically and in other pathogens, that evolutionary rates calculated from

outbreak data tend to be higher due to incomplete purifying selection (e.g. Gire et al., Science, 2014; Ho et al., MBE, 2005).

We have included a sentence in the manuscript highlighting this fact and citing Gire et al., Science, 2014; Ho et al., MBE, 2005:

“We obtained rates ranging from 2.74e-7 to 7.59e-7 substitutions/site/year (Table S3). Although these rates are ~9 times faster than previously calculated rates for the rice blast fungus (Gladieux et al., 2018; Latorre et al., 2022), it is expected that evolutionary rates calculated from disease outbreaks, such as the cases in Bangladesh and Zambia, are likely faster due to incomplete purifying selection (Gire et al., Science, 2014; Ho et al., MBE, 2005), and rates might vary in different blast fungus host-specific lineages”

I think increasing the MCMC chain length for convergence and altering the model/demographic would improve rates and dates.

As we have shown in this response, our new BEAST analysis using GTR for the substitution rate shows the robustness of our estimated evolutionary rates. Thus, the combination of four independent MCMC chains in our analysis proves to be sufficient to reach convergence with high ESS values (> 200) for all the estimated parameters, therefore we see no need to increase the MCMC chain length. Additionally, in the manuscript, we used a BEAST-independent approach that estimated very similar evolutionary rates.

All in all - this is a very good piece of research and must represent a huge effort from all involved, and I'd be happy to see this published.

We thank the reviewer again for her comments and suggestions.

Reviewer #2:
[identifies themself as Ping Wang]

Compared to Magnaporthe oryzae pathotype Oryza which causes the devastating rice blast, the M. oryzae pathotype Triticum infects wheat, causing

less known but economically important wheat blasts in geologically limited areas. However, due to world trade, the wheat blast has spread to South Asia and South Africa. Studies are urgently needed to monitor the transmission to avoid continued widespread impact on world food supplies. Here, Latorre and colleagues employed SNP analysis to examine the genotypes of various strains collected from the three continents to establish a single clonal linkage of the fungus. They also provided evidence demonstrating that the strains with the similar genetic backgrounds are controllable by host plants harboring the avirulent Rmg8 gene and by the fungicide Strobilurin. This is an excellent written study of significant importance. The conclusions are largely supported by experimental approaches and substantiated by statistical analysis.

I have no additional specific comments other than suggesting that the excellent review article entitled "Intercontinental Jumps and Its Management Strategies" by P. K. Singh and colleagues be included in the References.

We thank Reviewer 2 for his positive assessment of our manuscript.

We have now included the suggested reference in the introduction of our manuscript:

“The disease first appeared in 1985 in Brazil but has been reported in Bangladesh and Zambia over the last years (Singh et al., 2021).”

Reviewer #3:

Latorre et al. addresses a topic of broad interest and approaches the problem of introductions of potentially devastating crop pathogens from a number of important angles and represents a strong contribution to plant pathology. These angles include phylogenetic and population genetic analyses of a global collection of strains of the wheat blast fungus, genomic analyses of genes conferring virulence and antifungal drug resistance, tests of virulence and drug resistance, and mating ability. My only concern is about the claim of independence for the two introductions and my concern can be addressed by some straightforward additional analyses involving the current dataset and some

additional discussion. I have put my comments below quotes from the manuscript, which are preceded by @.

We thank Reviewer 3 for their positive assessment of our manuscript and their suggestions.

@Wheat, the most important food crop,

Some more information is needed. When I Google crops, rice comes up as the number one food crop.

To avoid a discussion about the criteria used to rank staple crops according to their importance (e.g. total cultivated area, human calorie intake, etc.), we have modified the sentence in the abstract:

“Wheat, one of the most important food crops, is threatened by a blast disease pandemic.”

@ following two independent introductions from South America

It is clear that there were two introductions, one to Bangladesh and one to Zambia, but it is not clear that they were independent. I see these possibilities. 1. One introduction from South America to Bangladesh and one from South America to Zambia. 2: One introduction from South America to Bangladesh and then an introduction from Bangladesh to Zambia. 3: One introduction from South America to Zambia and then an introduction from Zambia to Bangladesh. 4. The involvement of introductions to other, unsampled, regions of the globe. I do not think that you have enough data to settle on #1 and rule out the others. Number 4 is impossible to refute, but you should acknowledge it. Think about early publications on amphibian decline or Cryptococcus gatti that created scenarios to explain introductions that turned out to be wrong. You can determine if you have enough data to distinguish among scenarios numbers 1 - 3 by using trees constrained to reflect the scenarios and using likelihood ratio tests to see if your data are significantly more likely for one of them. My guess is that you won't be able to exclude both numbers 2 and 3. In which case, it would be best to consider the various scenarios and comment on how other data (historical records, etc.) support or refute each scenario.

The phylogenetic reconstruction we presented in the manuscript showed that Zambian (ZM) and Bangladeshi (BD) isolates are reciprocally monophyletic (with 100% bootstrap support and posterior probability of 1, for the Maximum Likelihood and the Bayesian reconstructions, respectively). This means that the two following statements are true: ZM isolates have a shared common ancestor that is more recent than the most recent common ancestor of any of the ZM isolates with any BD isolate; BD isolates have a shared common ancestor that is more recent than the most recent common ancestor of any of the BD isolates with any ZM isolate. Therefore, we favored scenario 1 in the manuscript: two independent introductions to Zambia and Bangladesh from South America. We thank the reviewer for pointing out scenario 4, which postulates that the introductions could have come from other unsampled location(s). We consider scenario 4 possible but unlikely. However, as the reviewer pointed out, scenario 4 is impossible to disprove. Therefore we have included a sentence in the main text acknowledging the possibility of scenario 4 (see quoted text below).

We think that the reciprocal monophyly of ZM and BD rules out by definition scenarios 2 and 3, introductions from Zambia to Bangladesh or from Bangladesh to Zambia, respectively. However, we have independently tested these two scenarios (2 and 3) against scenario 1 using constrained trees in IQ-TREE, as suggested by the reviewer. As expected, we found that the likelihood ratio test strongly favors scenario 1 over scenarios 2 and 3. We consider that this analysis is not relevant enough to be included in the manuscript.

Main text modification recognizing the possibility of scenario 4:

“We conclude that the clonal lineage has spread to Asia and Africa through at least two independent introductions, most probably from South America, although we cannot totally rule out that the source population was located in an unsampled location outside of South America.”

@cause total crop failure (5)

Are there data on the effect of wheat blast on the production of wheat in Bangladesh or Zambia? Reference 5 is about the synchrony of crop loss and not about wheat blast losses.

We have modified the statement about total crop failure and included a new sentence reporting the average yield loss caused by the wheat blast outbreak in, Bangladesh in 2016:

“In wheat, yield losses caused by pests and diseases average over 20% (4). Wheat is currently threatened by the expanding blast pandemic caused by the ascomycete fungus *Magnaporthe oryzae* (Syn. *Pyricularia oryzae*), a formidable and persistent menace to major grain cereals that could contribute to total crop failure (5).”

“The disease first appeared in 1985 in Brazil but has been reported in Bangladesh and Zambia over the last years, causing, for instance, an average yield loss of 51% in the Bangladesh outbreak in 2016.”

@twin challenge of climate change and armed conflicts in major agricultural regions.

Is there a reference for climate change or armed conflict and wheat blast? Or, one about the two problems and agricultural production in general?

We have included a reference that predicts how climate change will impact future yield gains by increasing disease risk (Chaloner et al, 2021, Nature Climate Change) and a reference directly referring to the impact of the Ukraine-Russia war on wheat production and food security (Bentley et al, 2022, Nature Food).

@However, the genetic identity and origin of the causal agent of an African outbreak, first detected in Zambia in 2018, remains unknown (8).

The authors cite reference 11 later in their ms regarding recombined regions of the wheat blast genome and this bioRxiv article also addresses the origin of the Zambian isolates. If there were any way to publish both this ms and that of ref.

11 back-to-back, science would be better served. Note that Latorre et al. goes well beyond reference 11 in testing virulence and drug resistance.

We do not see any conflict since we consider a bioRxiv preprint as a full publication. The publication of the preprint by Liu et al. (ref. 11) (June 19, 2022) was possible due to the data that our team made available to the community through our Zenodo preprint (March 25, 2021; https://doi.org/10.5281/zenodo.4637175). We are happy that their independent phylogenetic analysis reached the same conclusions that our manuscript, previously available as a biorXiv preprint (June 7, 2022; https://doi.org/10.1101/2022.06.06.494979).

@Here, we show that the recent emergence of wheat blast in Asia and Africa was caused by a single clonal lineage of the wheat blast fungus closely related to South American isolates and that the outbreaks in Zambia and Bangladesh originated by independent introductions.

See above comment about the independence of the introductions. It is also not clear to me that the introductions need to be single. Couldn't several closely related but genetically different strains have been involved in the introductions?

We addressed this point above and have modified the sentence in the main text accordingly. Notice the “at least” in the sentence, that includes now the possibility of multiple introductions from the same clonal lineage.

“We conclude that the clonal lineage has spread to Asia and Africa through at least two independent introductions, most probably from South America, although we cannot totally rule out that the source population was located in an unsampled location outside South America.”

@The B71 lineage shows reduced genetic diversity in comparison with South American isolates although incipient sub-structuring can be noted between Zambian and Bangladeshi clusters (Fig. 2A, inset).

Figure 7a and 7b show that much of the genetic variation in the Zambian clade may be due to the genome rearrangements and the mini-chromosome. I wonder if the authors have analyzed the amount of genetic variation and the timing of introductions after removing the non-clonal variation? If so, did it alter the story about the timing of introductions? If not, it would be worth doing to see if it does alter the timing.

Indeed, genetic variation in the Zambian clade might be due to structural rearrangements. Accordingly, we explained in the material and methods that "...the SNPs marked as putatively affected by recombination are preferentially located in genomic regions affected by structural variants, e.g. presence/absence variants. Such variants will generate phylogenetic discordances due to differential reference bias among the B71 isolates."

All the calculations of evolutionary rates and divergence times were carried out after removal of the non-clonal variation (e.g. the mini-Chromosome), as it can be seen if Fig. S8. This is explicitly stated multiple times in the Main Text and Material and Methods:

Main text:

"We leveraged the collection dates of *M. oryzae* clonal isolates to estimate their evolutionary rate. Before performing the tip-calibration analyses (12), **we removed regions that disrupt the clonal pattern of inheritance** (Fig. S6-S8) (13) and tested for a correlation between genetic distances and collection years (Fig. S9)."

Material and Methods:

We stated multiple times that we used only the recombination corrected tree.

"To test for the existence of a phylogenetic temporal signal (i.e. a positive correlation between sampling dates and genetic divergence) **we used the recombination-corrected tree generated by *ClonalFrameML*** (Fig. 2C)"

"As input for *BactDating*, **we used the recombination-corrected tree** generated by *ClonalFrameML*."

“From the alignment of the concatenated SNPs, **we masked those that *ClonalFramML* marked as putatively recombining** and used the masked alignment as input for the BEAST2 analyzes.”

@The B71 cluster is a clonal lineage.

This claim is solid.

We are glad to see that the reviewer agreed with the conclusion of our analysis.

@The B71 clonal lineage has recently expanded with independent introductions in Zambia and Bangladesh.

See comments above.

We addressed this point above.

@These findings are consistent with the conclusions of a recent independent study (11).

The Liu et al. bioRxiv publication advances a good phylogenetic argument for two, independent introductions, because the Bangladesh isolates are on a branch with a Brazilian isolate at the base and the Zambian isolates are on a branch subtended by a Bolivian isolate. Again, likelihood ratio tests would show if there are enough data to support this scenario over the others.

The tree presented by Liu et al, which the reviewer considers as a good phylogenetic argument, shows exactly the same topology as we present in Figure 2C. For this same reason we favored the hypothesis of two independent introductions to Zambia and Bangladesh from South America.

We addressed above the comment regarding the other possible scenarios suggested by the reviewer and the likelihood ratio tests.

@we removed regions that disrupt the clonal pattern of inheritance (Fig. S6-S8)

More explanation is needed here. Having just told the reader that the spread is clonal, you need to let the reader know how there can be non-clonal elements in the genome. Figure S7 needs to be described in more detail. This figure also provides an argument in favor of independent introduction because the Zambian isolates and the Bolivian isolate seem to share a mini-chromosome that is absent in the Brazilian and Bangladeshian isolates. This point is also made in the Liu et al. bioRxiv article.

The full explanation is available in the Material and Methods:

"Since the LD decay analyses revealed that the B71 pandemic lineage is a non-recombining clonal lineage, we hypothesized that the SNPs marked as putatively affected by recombination are preferentially located in genomic regions affected by structural variants, e.g. presence/absence variants. Such variants will generate phylogenetic discordances due to differential reference bias among the B71 isolates. To test this hypothesis we created full-genome alignments of the B71 and the 70-15 reference genomes using *Minimap2* (50) and visualized the output with *AliTV (51)*. Then, we overlapped the visual output with the SNPs putatively affected by recombination that were previously identified by *ClonalFrameML* (Fig. S9)."

We have modified the sentence in the main text to convey the same message presented in the material and methods:

"Before performing the tip-calibration analyses (12), we removed regions that disrupt the clonal pattern of inheritance (Fig. S6-S7) (13). Such regions were preferentially located in genomic regions affected by structural variants (Fig. S8), which results in phylogenetic discordances, even in clonal lineages, due to differential reference bias between B71 isolates and the reference genome."

@We scanned the available genomes and found that AVR-Rmg8 is conserved in all 36 isolates of the B71 clonal lineage even though the other 35 isolates of the Triticum lineage carry four diverse AVR-Rmg8

virulent alleles (eII, eII', eII'', eII''') that fully or partially evade immunity (Fig. 3A; Fig. S11) (16). B71 lineage isolates also lack the PWT4 effector, which is known to suppress AVR-Rmg8-elicited resistance (17).

This section of the ms is solid.

We are glad to see that the reviewer agreed with the conclusion of our analysis.

@These genome analyses predict that the B71 lineage isolates (AVR-Rmg8 positive, PWT4 negative) cannot infect wheat plants with the matching disease resistance gene Rmg8. To test this, we inoculated 14 B71 lineage isolates from Zambia and Bangladesh on wheat lines with and without the Rmg8 resistance gene (Fig. 3B; Fig. S12). Unlike a distinct South American isolate, none of these pandemic isolates could infect Rmg8 wheat plants.

This section of the ms is solid.

We are glad to see that the reviewer agreed with the conclusion of our analysis.

@Remarkably, all but one Brazilian isolate (12.1.181) of the 36 B71 lineage genomes carry the G1243C allele and are predicted to be strobilurin sensitive. We tested this by assaying B71 lineage isolates and found that all tested 30 isolates are strobilurin sensitive (Fig. 4B-C; Fig. S13).

From the legend to Figure 4b-c, it seems that only the Zambian isolates were tested for antifungal resistance.

We thank the reviewer for identifying a typo in the figure legend of Figure 4B-C. The legend should have stated that all Zambian and Bangladeshi isolated were found to be “strobilurin susceptible”. See amended figure legend below:

“...indicating that all the Zambian and Bangladeshi isolates have the ‘strobilurin susceptible’ genotype as anticipated by their *CYTB* sequences.”

If strain 12.1.181 has the genome of a strobilurin resistant strain, and it is basal to the Bangladesh clade, are all Bangladesh strains resistant?

No. All the Bangladeshi strains we tested are strobilurin susceptible. We have changed the legend of Figure 4B-C.

How did susceptible strains emerge from a population of resistant strains? This point raised the possibility of other populations of wheat blast in other global locations. Note in Figure 4b that there is some phylogenetic distance between the Bangladesh or Zambia populations and their closest South American relative.

Fungicide resistance to strobilurin can appear de novo as shown by our own experiment:

“In laboratory experiments, we could readily recover spontaneous strobilurin (azoxystrobin) resistant mutants of African isolate ZMW20-14 (Fig. 4B-C) consistent with a high potential for emergence of fungicide resistance in the pandemic clonal lineage.”

Additionally, based on phylogenetic inferences, we and others have shown that resistance to strobilurin has emerged multiple times (Castroaguadin et al, 2015) (ref. 18).

All data appear to be available to the scientific community.

We are glad to see that the reviewer acknowledges our commitment to open and reproducible science.
